# Role of Microalbuminuria and Hypoalbuminemia as Outcome Predictors in Critically Ill Patients

**DOI:** 10.1155/2021/6670642

**Published:** 2021-04-12

**Authors:** Mahmoud Nour, Abdelhaleem hegazy, Abeer mosbah, Ahmed Abdelaziz, Mohamed Fawzy

**Affiliations:** ^1^Critical Care Medicine Department, Cairo University, Giza, Egypt; ^2^Clinical Pathology Department, Mansoura University, Mansoura, Egypt

## Abstract

**Background:**

Assessment of microalbuminuria and hypoalbuminemia can be used as a good tool for the prediction of the ICU outcome in critically ill patients.

**Purpose:**

To evaluate and compare the prognostic significance of microalbuminuria (albumin creatinine ratio (ACR)) and serum albumin level done on admission and after twenty-four hours for the critically ill patients. *Methodology*. Sixty ICU patients were involved in a prospective cohort study (mean age was 44.4 ± 16.7 years, and 78.3% were males). Patients were divided into 2 groups according to mortality (survivors and nonsurvivors) and were subjected to laboratory measurement of the mentioned biomarkers on admission and after twenty-four hours.

**Results:**

There were 34 patients (56.67%) in group A (survivors) and 26 patients (43.33%) in group B (nonsurvivors). Albumin creatinine ratio on admission (ACR1) and albumin creatinine ratio after 24 hours (ACR2) were significantly lower in survivors than nonsurvivors (*P* values were <0.001 for both). Serum albumin level after 24 hours of admission was significantly higher in survivors than nonsurvivors (*P* value 0.02) while admission serum albumin was not significantly different between both groups (*P* value was 0.1). There was a positive correlation between ACR2 and ICU stay and mechanical ventilatory support with a strong positive correlation with the use of vasopressor therapy (*r*: 0.35, 0.58, and 0.73, respectively). *P* values were 0.005, <0.0001, and <0.0001, respectively. There was a positive correlation between ACR2 with APACHE II and SOFA scores (*r*: 0.46 and 0.43, respectively); *P* values were 0.001 and <0.0001, respectively. There was a moderate negative correlation between serum albumin on admission and after 24 hours and the duration of mechanical ventilation (*r*: −0.4 and −0.39, respectively) (*P* values were 0.001 and 0.002, respectively). By Cox regression analysis, two parameters were found to be an independent predictor of mortality in ICU patients which were age and using vasopressor treatment (*P* values = 0.01 and <0.001), while the other parameters were not independent predictors of mortality (*P* values were more than 0.05).

**Conclusions:**

Microalbuminuria on admission and after 24 hours of ICU admission could be a good predictor of mortality in critically ill patients. The serum albumin level after 24 hours of admission can predict poor outcomes in critically ill patients.

## 1. Introduction

The predictive power of microalbuminuria for renal and cardiovascular disease morbidity and mortality was subsequently confirmed in type 2 diabetes, arterial hypertension, and the general population [1, 2].

Isolated spot measurements of microalbuminuria may be misleading and less reliable than the rate of change of microalbuminuria, specifically a rise in albumin excretion rate, as an indicator of progressive renal disease. Moreover, some patients apparently can develop renal impairment without developing microalbuminuria [3–6].

There is no proven early histological marker of progressive renal disease in diabetes; an overwhelming body of evidence indicates that a small increase of albumin in the urine above the normal range is one of the strongest predictive biomarkers of cardiorenal disease [7–9]. Of course, the level of risk increases further as the albuminuria gets heavier, but this represents the late phases of the disease. Whether microalbuminuria or for that matter albuminuria is on the causal pathway for cardiorenal disease and is thus a surrogate for it still remains an open question [10].

Microalbuminuria, defined as 30–300 mg/day of albumin excretion in the urine, occurs rapidly after an acute inflammatory insult such as sepsis and persists in patients with complications [11–13].

It is a common finding in critically ill patients, where it has shown promise as a predictor of not only organ failure and vasopressor requirement but also of mortality, faring better than Acute Physiological and Chronic Health Evaluation (APACHE) II score and Sequential Organ Function Assessment (SOFA) scores in some studies [14, 15].

The optimal use of intensive care unit (ICU) resources and an accurate method of predicting the outcome of the critically ill has been the aim of many studies over several years [16]. The postoperative period may be more problematic in diabetic patients with microalbuminuria, but microalbuminuria does not seem to have a major effect on the postoperative course in patients undergoing CABG [17].

Microalbuminuria is a reflection of capillary leak, which is observed in multiple organ dysfunction syndrome [18]. In this syndrome, diffuse systemic inflammation results in a systemic capillary leak in most vascular beds, including the kidney, where peak albuminuria is observed in patients with systemic inflammation up to 2 days before a detectable rise in other markers of inflammation [19]. Microalbuminuria has shown promise as an early predictor of disease severity in many acute inﬂammatory conditions [19]. More importantly, it has been found to be predictive of mortality in a heterogeneous group of critically ill patients [20]. Several studies have described a rapid increase in urine albumin (microalbumin) excretion in acute inﬂammatory conditions, which appears to be related to systemic vascular damage exempliﬁed by capillary leak [21].

### 1.1. Aim of Work

The objective of the study is to evaluate if the presence of microalbuminuria and hypoalbuminemia could be correlated with the clinical outcome in critically ill patients.

### 1.2. Patients and Methods

Our study was a prospective and comparative study conducted as a single-center study including 60 consecutive adult patients who were admitted to the Critical Care Medicine Department, Cairo University.

#### 1.2.1. Inclusion Criteria

All adult patients ≥18 years old admitted to our ICU for more than 24 hours were included in the study.

#### 1.2.2. Exclusion Criteria

Exclusion criteria are as follows: the presence of confounding factors such as anuria, urinary tract infection, macroscopic hematuria, preexisting chronic kidney disease, pregnancy, disseminated malignancy, and female patients with menstruation.

#### 1.2.3. Patient Demography

On admission, the following data was collected for each patient: age, weight, body mass index (BMI), gender, date and time of admission, patient's clinical classification (medical or surgical), provisional diagnosis, and comorbid conditions such as diabetes, hypertension, and heart failure.

#### 1.2.4. Evaluation of Patients

All included patients were subjected to the following: full clinical evaluation including history and physical examination with special emphasis on vital signs (blood pressure, heart rate, temperature, and respiratory rate) and central venous pressure. All data was recorded at the time of admission. Urine output (UOP/hr, UOP/day) and total balance were recorded in the first 24 hours of admission.

For disease severity scoring, APACHE II and SOFA scores were calculated from data collected during the first 24 hrs following ICU admission. [22, 23]. Each patient was followed up throughout their ICU stay for a maximum of 28 days, and the following outcome data were obtained: ICU length of stay, duration of mechanical ventilation and ventilation free days, need for vasopressor use, acute renal failure, and need for continuous renal replacement therapy and mortality.

#### 1.2.5. Laboratory Investigations

Routine laboratory tests were investigated: CBC (complete blood count), coagulation profile: PT, INR, and PTT, ABGs (arterial blood gases), liver function tests (LFTs), and kidney function test.

Specific Laboratory Measurements: spot urine samples were collected by ICU nurses within 8 hours of ICU admission and again at 24 hours from admission, microalbumin and creatinine from these urine samples were analyzed, and we used urinary albumin to creatinine ratio (ACR) to correct for variations in urinary ﬂow rate. The samples which were collected on admission were referred to as ACR1 and those samples which were collected after 24 hours of ICU admission were referred to as ACR2; then, urine samples were received by the biochemistry laboratory and stored at 2–8°C till analysis.

Urinary microalbumin was measured using the immunoturbidimetric method (Dimension RxL Max, Dade Behring Inc., USA). Urinary creatinine was measured using a modified kinetic Jaffe reaction (Dimension RxL Max, Dade Behring Inc., USA).

Microalbuminuria, expressed as ACR in mcg/mg, is defined as ACR of less than 300 mcg/mg. The ratio has a conventional cutoff value of 30 mcg/mg in the healthy reference population. The reference range for mortality prediction in a critically ill population is yet to be determined. [24].

Freshly collected blood samples were used to detect serum albumin levels on ICU admission and after 24 hours of ICU admission were collected for quantification of albumin in human serum which were referred to as serum albumin 1 and serum albumin 2, respectively (using dimension clinical chemistry system).

## 2. Evaluation of Outcome Data

We evaluated the following: the length of ICU stay (LOS), 28-day mortality, and organ supportive measures (hemodialysis, mechanical ventilation, and using vasopressor/inotropic medications). We divided the studied population into two groups according to mortality: survivors (group A) and nonsurvivors (group B), and the results were compared in both groups.

### 2.1. Statistical Analysis

The completed data were coded, processed, and analyzed through SPSS (Statistical Package for Social Sciences) (Standard version release 20.0). Descriptive statistics in the form of frequencies and percentages were used for qualitative data. Chi-Square was used for testing the significance of discrete and categorical variables. Mean and standard deviation was used to summarize parametric continuous variables. Median, minimum, and maximum were used to summarize nonparametric continuous variables. An independent *t*-test was used to compare between two means.

However, Mann–Whitney *U* test was used to compare continuous nonparametric variables in 2 different groups. Discrimination between hospital survivors and nonsurvivors was evaluated by receiver operating characteristic (ROC) curve analysis. Kaplan–Meier survival analysis was done, and the Log Rank test was used to test for equality of survival distributions among different levels of independent variables. The correlation coefficient was used to illustrate the strong relationship between variables. Significant predictors in the bivariate analysis were entered into the regression model. Odds ratios and their 95% confidence interval were calculated. *P* ≤ 0.05 was considered as the level of statistical significance.

## 3. Results

The study was conducted on 60 patients admitted to the Critical Care Department, Cairo University, from September 2013 to February 2015.

The results of the present work will be described under the following headings:

1. Demographic and clinical data
2. Laboratory data
3. Outcome data
4. Statistical correlations

### 3.1. Demographic and Clinical Data

Baseline demographic data regarding gender and comorbid conditions (DM, HTN) were recorded, most of the patients were males (47 patients) (78.3%), 17 patients (28.3%) had diabetes mellitus, and 13 patients (21.7%) had hypertension (HTN).

The current study showed that the mean age of the studied population was 44.4 ± 16.7 years, the mean height was 170.9 ± 8.8 cm, the mean weight was 76.6 ± 14.7 kg, and the mean body mass index (BMI) was 26.2 ± 4.5 kg/m^2^, and 40 patients (66.67%) were admitted for nonsurgical causes.

#### 3.1.1. ICU Mortality

The 28-day mortality was encountered in 26 patients which represents 43.3% of the whole study population. The studied patients were divided into two groups according to 28-day mortality (survivors and nonsurvivors).

#### 3.1.2. Comparative Results of Both Groups

The study populations were divided into 2 groups: group (A) survivors which included 34 patients (56.67%) and group (B) nonsurvivors which included 26 patients (43.33%) (Table 1).

Regarding gender distribution, 29 patients (85.3%) were males in group A versus 18 patients (69.2%) in group B with no statistically significant difference between both groups, while regarding age, there was a significant difference between both groups: group A had a mean age of 37.3 ± 14.6 years while group B had a mean of 53.8 ± 14.7 years (*P* value <0.001). Regarding height, group A had a mean height of 171.5 ± 9.08 cm and group B had a mean height of 170.35 ± 8.5 cm with no statistically significant difference. Also, there was no statistically significant difference between both groups regarding weight; group A had a mean weight of 74.68 ± 16.5 kg, and group B had 79.19 ± 11.8 kg (*P* value was 0.2) (Table 1).

The current study showed no significant difference between both groups regarding BMI: group A had a mean BMI of 25.5 ± 5.4 kg/m^2^ and group B had 27.2 ± 2.6 kg/m^2^, while the prevalence of DM and HTN showed significant differences between both groups; for DM, group A had 5 patients (14.7%) and group B had 12 patients (46.2%) (*P* value was 0.007), and for HTN, group A had 4 patients (11.8%) and group B had 9 patients (34.6%) (*P* value was 0.03) (Table 1).

### 3.2. Laboratory Data

The present work showed statistically significant differences between survivors (A) and nonsurvivors (B) regarding albumin creatinine ratio on ICU admission (ACR1), albumin creatinine ratio after 24 hours of ICU admission (ACR2), and serum albumin level after 24 hours of ICU admission (serum albumin 2), but for serum albumin level on ICU admission (serum albumin 1), there was no statistically significant difference (Table 2).

### 3.3. Outcome Data

The current study showed statistically significant differences between survivors and nonsurvivors:

  (A) The median ICU stay was 7 days [4–21] in survivors while it was 10 days [3–23] in nonsurvivors, (*P* value 0.002) (Table 3)
  (B) Need for vasopressor (norepinephrine) and inotropes (dobutamine): 10 patients (29.4%) needed their support in group A compared to 24 patients (92.3%) in group B (*P* value was <0.001) (Table 3)
  (C) The mean duration of mechanical ventilation: in survivors, it was 3.5 days while in nonsurvivors it was 9 days (*P* value was <0.001) (Table 3)
  (D) Incidence of acute kidney injury (AKI) and need for continuous renal replacement therapy (CRRT): two patients (5.9%) in group A had AKI and seventeen patients (65.4%) in group B (*P* value < 0.001); also, CRRT was used in one patient (2.9%) in the survivor group and was used in 15 patients (57.7%) in nonsurvivor, (*P* value < 0.001) (Table 3)

In the current study, 43 patients were nondiabetics, 47 patients were nonhypertensives, and 40 patients showed no history of hypertension nor diabetes.

On studying this subgroup of the study, we found the followings:

-The total number of patients without a history of diabetes mellitus or hypertension was 40
-Out of those 40 patients, 29 patients survived while 11 patients died
-On comparing the survivors and nonsurvivors of this subgroup, we found no statistically significant difference regarding the mean BMI (23.6 versus 24.8 Kg/m^2^, respectively, with *P* value of 0.36), yet, there was a statistically significantly lower mean age in survivors as compared to nonsurvivors (39.6 versus 56.4 years, *P* value < 0.001)
-The present study showed a statistically significant difference between survivors and nonsurvivors in this nondiabetics and nonhypertensive subgroup:
    1: mean albumin/creatinine ratio on admission (ACR1) was statistically significantly lower in survivors when compared to nonsurvivors (30.6 versus 126 ug/mg, *P* value < 0.001)
    2: mean albumin/creatinine ratio after 24 hours of admission was statistically significantly lower in survivors when compared to nonsurvivors (32.4 versus 144.4 ug/mg,*P* value < 0.001)
    3: mean serum albumin level on admission (albumin 1) was statistically significantly higher in survivors compared to nonsurvivors (3.2 versus 2.62 gm/dl respectively, *P* value 0.01)
    4: mean serum albumin level after 24 hours of admission (albumin 2) was significantly higher in survivors compared to nonsurvivors (2.94 versus 2.55 gm/dl respectively, *P* value 0.03)
On studying the outcome parameters in this subgroup of the study population, we found a statistically significantly lower mean duration of ICU stay, lower need for vasopressor therapy (norepinephrine), lower need for renal replacement therapy, and lower mean duration of mechanical ventilation in survivors compared to nonsurvivors (6 versus 11 days, *P* 0.004, 10% versus 100%, *P* value < 0.0001, 0% versus 45.6%, *P* value< 0.001, 4.2 versus 8.8 days, *P* value <0.001, respectively).

According to ACR1 at a cutoff point of 125 mcg/mg, the survival analysis has been performed utilizing Kaplan–Meier, and patients with ACR less than 125 mcg/mg on admission stayed in ICU for 8.27 days versus 11.65 days for patients with ACR of more than 125 mcg/mg (*P* value 0.009).

According to ACR 2 at a cutoff point of 125 mcg/mg, the survival analysis has been performed utilizing Kaplan–Meier, and patients with ACR2 less than 125 mcg/mg stayed in ICU for 15.5 days versus 12.8 days for patients with ACR2 of more than 125 mcg/mg, with no statistically significant difference (*P* value 0.2).

According to serum albumin on admission at a cutoff point of 2.5 g/dl, the survival analysis has been performed utilizing Kaplan–Meier, and patients with serum albumin of less than 2.5 g/dl stayed in ICU for 14.122 days versus 13.596 days for patients with serum albumin of more than 2.5 g/dl (*P* value was not significant, 0.71).

According to serum albumin after 24 hours of admission at a cutoff point of 2.5 g/dl, the survival analysis has been performed utilizing Kaplan–Meier, and patients with serum albumin of less than 2.5 g/dl stayed in ICU for 9.96 days versus 8.9 days for patients with serum albumin of more than 2.5 g/dl (*P* value was insignificant, 0.3).

When we used serum albumin at a cutoff of 2.5 g/dl as a tool for predicting the mortality in ICU patients, we found AUC was 0.6 and 0.64 for ICU admission and after 24 hours of ICU admission, respectively, with a sensitivity of 42%, specificity of 74% (for serum albumin 1), sensitivity of 57%, and specificity of 62% (for serum albumin 2) (*P* value was insignificant, 0.930) (Table 4).

### 3.4. Predictors of ICU Outcome

ACR1, ACR 2, serum albumin 1, serum albumin 2, APACHE II, and SOFA scores were tested as predictors for ICU outcome. By using Cox regression analysis, these markers were not statistically significant predictors for ICU mortality; *P* values were 0.431, 0.556, 0.719, 0.930, 0.6260, and 412, respectively.

In the current work, by using logistic regression analysis, we found that age and using vasopressors were significant predictors of mortality in critically ill patients (*P* values were 0.01 and <0.001, respectively).

**Figure 1:**
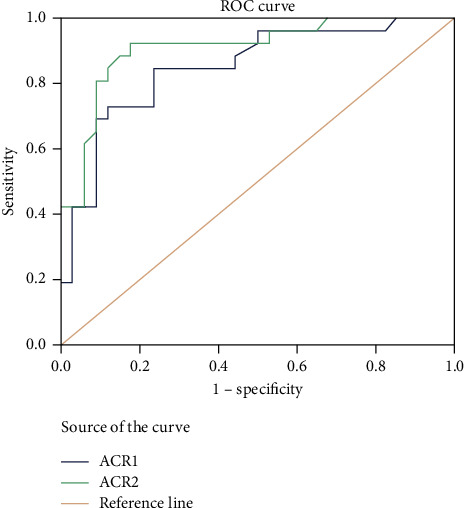
ROC curve analysis of ACR1 and ACR2 as prognostic markers of ICU mortality.

**Figure 2:**
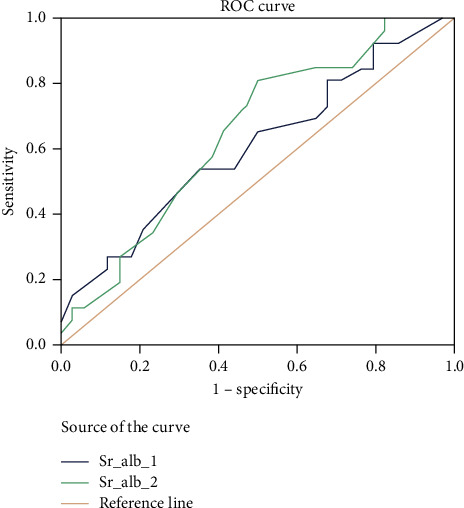
ROC curve analysis of serum albumin 1 and serum albumin 2 as prognostic markers of ICU mortality.

For the entire study population, the area under the ROC curves for mortality was the highest for ACR 2 (0.91) (Figure 1), followed by ACR1 (0.85) (Figure 1) then SOFA score (0.69), APACHE II (0.68), then serum albumin 1 (0.64) (Figure 2), and lastly serum albumin 2 (0.60) (Figure 2).

## 4. Discussion

In the present study, among all the patients admitted to ICU, microalbuminuria was found to be prevalent in a broad spectrum of critically ill patients, 91.6% of patients had ACR >30 mcg/mg on ICU admission, and it persisted in 86.6% at 24 hours of ICU admission while after 24 hours of admission to ICU, 41.6% of patients had ACR levels of more than 100 mcg/mg.

These results were more nearly similar to a study done by Basu et al. [25]; in his study of 238 critically ill patients, they found that on admission, 76% of patients had ACR > 30 mcg/mg on ICU admission, and it persisted in 67% at 24 hours. At 24 hours, 43% of patients had ACR levels of more than 101 mcg/mg.

Lower results were shown by Patel et al. [26]; 71% of patients had ACR > 30 mcg/mg on ICU admission, and it persisted in 68% at 24 hours of ICU admission. At 24 hours, 34% of patients had ACR levels of more than 101 mcg/mg.

On admission, microalbuminuria was found to be significantly elevated in nonsurvivors as compared to survivors: 141 mcg/mg (31–1014) and 51.5 mcg/mg (5–272), respectively with *P* < 0.001. Later after 24 hours, ACR2 was significantly elevated in the nonsurvivors 175 mcg/mg versus 49 mcg/mg in survivors (*P* < 0.001).

Patel et al. [26] had similar results; the median ACR1 was also higher significantly in nonsurvivors (161.5 (IQR 105.3–180.8) mcg/mg) than survivors (89.8 (IQR 28.7–101.3 mcg/mg) (*P* < 0.001). The median ACR2 was significantly higher in the patients who died in the ICU (164.5 (IQR 104.9–172.1 mcg/mg) in comparison to those who survived (46.0 (IQR 25.6–89.4) mcg/mg) (*P* < 0.0001).

Bhadade et al. [27] found similar results; the median levels for ACR1 were 152.70 mcg/mg (IQR 108.71 to 194.92) and 44.48 mcg/mg (IQR 26.80 to 108.41) for the sepsis and nonsepsis groups, respectively. The levels of microalbuminuria were significantly high among the patients with sepsis at admission as compared to those without sepsis. These levels continued to remain significantly high among the nonsurvivors, whereas they had dropped among those who survived.

Bhadade et al. [27] found that those who died (37 patients) had a median ACR1 of 172.98 mcg/mg which increased to 246.22 mcg/mg after 24 hours. Similar findings were echoed in a study done by Basu et al. [25].

In our study, there was a reduction in ACR after 24 hours in survivors, which was in accordance with previous studies of Abid et al. [28] and Gosling et al. [29] indicating that failure of ACR to decline is associated with increased ICU mortality. We found that nonsurvivors had higher levels of microalbuminuria on ICU admission compared to survivors. Also, there was a significant increase in microalbuminuria in nonsurvivors at 24 hrs.

This finding goes hand in hand with a previous study of Mackinnon et al. [20] who did a pilot study and examined a heterogeneous group of patients (around 60 patients) with a variety of clinical conditions, which accurately reflected patients admitted to a general ICU in the UK. This was considered advantageous, as any predictive test result must be applicable to all patients with a wide variety of diagnoses, not just small highly selected groups.

A cutoff value of 125 mcg/mg was chosen in the current study as it is more than 4 times the lower border for microalbuminuria detection to increase the power of prediction of outcome. We found that 33.3% of our patients had a level of more than 125 mcg/mg with a positive predictive value (PPV) of 85% and a negative prediction (NPV) of 77.5%; these patients were five times more likely to die compared to microalbuminuria of less than 125 mcg/mg on admission.

Our results were similar to those done by Basu et al. [25], where at a cutoff of 124 mcg/mg, the sensitivity was 80%, specificity was 64%, a positive predictive value (PPV) was 51%, and a negative predictive value (NPV) was 87%.

Basu et al. [25] and Gosling et al. [29] observed that urine microalbumin increased rapidly within 6 hours following ICU admission and predicted ICU mortality. Abid et al. [28] reported the positive predictive value of increasing microalbuminuria (ACR) to differentiate MODS from other categories of sepsis to be 50% and negative predictive value as 96%.

The current study showed that ACR2 had the highest prediction of mortality with an AUC curve of 0.91. Similar to our work, Patel et al. [26] showed that with receiver operating characteristic curve (ROC) analysis, ACR2 merged as the best indicator of mortality (AUC of ACR2 = 0.85 > AUC of ACR1 = 0.81>).

On the other hand, other studies found that ACR on admission is more predictive than that after 24 hours of admission. In a study of 50 patients in a heterogeneous ICU population, Mackinnon et al. [20] calculated the probability of death for ACR and suggested that a rapid indication of the outcome can be obtained within 6 h of ICU admission.

We speculated that ACR1 did not correlate well with the outcome in our study like others as most of our patients were not admitted directly to the intensive care unit, but from the emergency room and the ward where they were initially stabilized. Hence, the ACR measured on admission to our ICU is not a true reﬂection of early ACR elevation. In addition to this, most of the previous studies were carried on septic patients while our study was carried on the general ICU population.

A recent study by Byron et al. [30] done over 92 patients suggested that point of care (POC) ACR could help to rapidly identify Emergency Department (ED) sepsis patients without obvious hemodynamic compromise who may benefit from aggressive monitoring and resuscitation.

In our study, we found that hypoalbuminemia after 24 hours of admission was significantly different between survivors and nonsurvivors (*P* value 0.02) but there was no significant difference between both groups on admission (*P* value was 0.1). We made a cutoff level of 2.5 mg/dl as a predictor of outcome; the ROC curve analysis revealed that measurements of serum albumin on admission and after 24 hours were 0.6 and 0.64, respectively, there was a moderate negative correlation between serum albumin 1 and 2 and the duration of mechanical ventilation (−0.4 and −0.39, respectively). As regards the mean survival time, serum albumin was not a significant predictor. *P* value was 0.3. This may be due to the small size of our study.

Contrary to our findings, the following studies showed a strong prediction of hypoalbuminemia to mortality; the main difference between our study and their studies is the huge number of the studied population in these studies. In a large study (including 5451 patients) done by Jellinge et al. [31], they found that hypoalbuminemia correlated strongly with 30-day mortality. Also, they stated that hypoalbuminemia had a good negative predictive correlation with 30-day mortality. Thus, they suggested that the emergency physician could use plasma albumin as a predictive marker when assessing patients upon arrival to the Emergency Department.

Our work has some limitations. The small-sized sample made some results not significant. While it is true that many conditions such as age (>40 yrs), smoking, diabetes mellitus, and hypertension are independent causes of microalbuminuria in the general population, these patients were included, since their exclusion would have made the study population less representative of the real critical illness scenario. Moreover, by choosing a 4 times higher cutoff value of ACR (125 mcg/mg for the mortality), the high NPV of the test could rule out most patients who had elevated ACR (by the conventional cutoff of 30 mcg/mg) due to confounders.

## 5. Conclusion

Microalbuminuria is of common occurrence in a heterogeneous critically ill population. At 24 hours, the absence of elevated levels of microalbuminuria is strongly predictive of ICU survival. Microalbuminuria is an inexpensive and rapid diagnostic tool; serial measurements may prove a useful aid in the clinical assessment of critically ill patients at risk of worse prognosis, even in resource poor areas. Microalbuminuria at 24 hours of ICU admission is a good predictor of mortality (like APACHE II score) in critically ill patients. Age and using vasopressor support had a high prediction of mortality in critically ill patients. Serum albumin level can predict the poor outcome but this needs large multiple center studies to determine the threshold reference value.

## Figures and Tables

**Table 1:** Comparative demographic and clinical data in the two study groups.

Group	(A) survivor *N* = 34	(B) nonsurvivor *N* = 26	*P* Value
Gender;			
Male no, (%)	29 (85.3)	18 (69.2)	0.13
Female no, (%)	5 (14.7)	8 (30.8)	
Age (years) Mean ± SD	37.3 ± 14.6	53.8 ± 14.7	**<0.001**
Height (cm) Mean ± SD	171.5 ± 9.08	170.35 ± 8.5	0.6
Weight (kg) Mean ± SD	74.68 ± 16.5	79.19 ± 11.8	0.2
BMI (kg/m^2^) Mean ± SD	25.5 ± 5.4	27.2 ± 2.6	0.15
DM (mg/dl) no, (%)	5 (14.7)	12 (46.2)	**0.007**
HTN (mm Hg) no, (%)	4 (11.8)	9 (34.6)	**0.03**

BMI: body mass index; SD: standard deviation; DM: diabetes mellitus; HTN: hypertensive disease.

**Table 2:** Laboratory data in the two study groups.

Laboratory data	(A) Survivor *N* = 34	(B) Nonsurvivor *N* = 26	*P* Value
ACR 1 mean; (IQR)	51.5 mcg/mg (5–272)	141.00 mcg/mg (31–1014)	**<0.001**
ACR 2 mean; (IQR)	49 mcg/mg (4.6-220)	175 mcg/mg (35–357)	**<0.001**
Serum albumin 1 mean ± SD	3.0 g/dl (0.7)	2.7 g/dl (0.7)	0.1
Serum albumin 2 mean ± SD	2.8 g/dl (0.5)	2.5 g/dl (0.4)	**0.02**

ACR1: albumin creatinine ratio on admission; ACR2: albumin creatinine ratio after 24 hours; serum albumin 1: on admission; serum albumin 2: after 24 hours; SD: standard deviation; IQR: interquartile range.

**Table 3:** Outcome data in the two study groups.

Variables	(A) Survivors *N* = 34	(B) Nonsurvivors *N* = 26	*P* value
ICU stay (days) median (IQR)	7 (4–21)	10 (3–23)	0.002
Use of vasopressor (no. & (%))	10 (29.4%)	24 (92.3%)	<0.001
Duration of mech. vent. (days) median (IQR)	3.5 (0–13)	9 (3–23)	<0.001
AKI	2 (5.9%)	17 (65.4%)	<0.001
CRRT	1 (2.9%)	15 (57.7%)	<0.001

IQR: interquartile range; mech. vent.; mechanical ventilation; AKI: acute kidney injury; CRRT: continuous renal replacement therapy.

**Table 4:** Performance of serum albumin 1 and serum albumin 2 as predictors for the mortality in ICU patients.

Variables	Area			Validity	
				Sensitivity (%)	Specificity (%)
Serum albumin 1	0.60	0.07		42	74
Serum albumin 2	0.64	0.07	2.5 g/dl	57	62

## Data Availability

According to our institutional policies, the study data should not be freely available and its access is restricted.
